# Pediatric in-Hospital Death from Infectious Disease in Uganda: Derivation of Clinical Prediction Models

**DOI:** 10.1371/journal.pone.0150683

**Published:** 2016-03-10

**Authors:** Nasim Lowlaavar, Charles P. Larson, Elias Kumbakumba, Guohai Zhou, J. Mark Ansermino, Joel Singer, Niranjan Kissoon, Hubert Wong, Andrew Ndamira, Jerome Kabakyenga, Julius Kiwanuka, Matthew O. Wiens

**Affiliations:** 1 Department of Anesthesiology, Pharmacology, & Therapeutics, University of British Columbia, Vancouver, Canada; 2 Center for International Child Health, BC Children’s Hospital, Child and Family Research Institute, Vancouver, Canada; 3 Department of Pediatrics, Mbarara University of Science and Technology, Mbarara, Uganda; 4 Department of Statistics, University of British Columbia, Vancouver, Canada; 5 School of Population and Public Health, University of British Columbia, Vancouver, Canada; 6 Canadian HIV Trials Network, St. Paul’s Hospital and University of British Columbia, Vancouver, Canada; 7 Department of Pediatrics, BC Children’s Hospital and University of British Columbia, Vancouver, Canada; 8 Maternal, Newborn and Child Health Institute, Mbarara University of Science and Technology, Mbarara, Uganda; University College London, UNITED KINGDOM

## Abstract

**Background:**

Pediatric hospital mortality from infectious diseases in resource constrained countries remains unacceptably high. Improved methods of risk-stratification can assist in referral decision making and resource allocation. The purpose of this study was to create prediction models for in-hospital mortality among children admitted with suspected infectious diseases.

**Methods:**

This two-site prospective observational study enrolled children between 6 months and 5 years admitted with a proven or suspected infection. Baseline clinical and laboratory variables were collected on enrolled children. The primary outcome was death during admission. Stepwise logistic regression minimizing Akaike’s information criterion was used to identify the most promising multivariate models. The final model was chosen based on parsimony.

**Results:**

1307 children were enrolled consecutively, and 65 (5%) of whom died during their admission. Malaria, pneumonia and gastroenteritis were diagnosed in 50%, 31% and 8% of children, respectively. The primary model included an abnormal Blantyre coma scale, HIV and weight-for-age z-score. This model had an area under the curve (AUC) of 0.85 (95% CI, 0.80–0.89) with a sensitivity and specificity of 83% and 76%, respectively. The positive and negative predictive values were 15% and 99%, respectively. Two alternate models with similar performance characteristics were developed withholding HIV and weight-for-age z-score, for use when these variables are not available.

**Conclusions:**

Risk stratification of children admitted with infectious diseases can be calculated based on several easily measured variables. Risk stratification at admission can be used for allocation of scarce human and physical resources and to guide referral among children admitted to lower level health facilities.

## Introduction

The fourth United Nations Millennium Development Goal (MDG4) aims to reduce global under five mortality by two thirds from the levels seen in 1990 before the end of 2015.[1] Despite substantial worldwide progress and significant gains in some regions, this achievement will not be reached in most countries of sub Saharan Africa.[2] Currently, over six million children under five years of age continue to die annually, most from infectious diseases.[3,4]

Severe infections including pneumonia, malaria and diarrhea remain the most common cause of death in children worldwide. Pneumonia alone kills more than 1.2 million children annually in resource constrained countries.[4,5] To help address this burden, the World Health Organization introduced the Integrated Management of Childhood Illness (IMCI) guidelines offering hospitals and health centers a syndrome based approach to identify the major causes of death in children under five.[6] Whereas the IMCI guidelines provide an approach to the identification and management of common pediatric diseases, they do not provide any risk scores for the identification of children at high risk of mortality. While the IMCI guidelines do provide a list of danger signs for each condition, facilitating an approach for referral decisions, these were not developed for risk scoring *per se*.

Several risk scoring models have been developed for use in developed countries to determine in-hospital mortality in pediatric patients. The Pediatric Risk of Mortality (PRISM), Pediatric Index of Mortality (PIM) and (PIM2), are models that predict mortality in children admitted to a pediatric intensive care units.[7–9] These models calculate risk based on several physiologic characteristics and can help identify children requiring more focused care. In resource constrained countries efficient resource utilization is especially important and models that can effectively predict risk could be used to facilitate such prudent resource utilization. This may be especially important for children admitted to lower-level health facilities where the capacity to treat critically ill children is absent or limited. In this context, risk scores could play a crucial role in guiding referral to higher level health centers and hospitals which may be better equipped to care for these children. Models such as PRISM and PIM were derived in developed countries and do not take into consideration major differences seen in children from resource constrained countries, in particular the high prevalence of HIV and malnutrition. Health system limitations for diagnosis, treatment and referral, and social factors such as the prevalence of poor health seeking behavior are also substantially different. Further, the criteria used in the application of these models (base excess, FiO2, time to ventilation etc.) are often not available or relevant in a resource constrained context where mechanical ventilation and modern critical care techniques are the exception. New models developed in and for a resource constrained environment are urgently needed. Where these models have been evaluated in resource constrained countries, it has been done in environments atypical to what is generally available in this context. Recently, a risk scoring algorithm for prediction of in-hospital mortality in subjects with respiratory disease has been developed for use in more a typical East African resource constrained context.[10] However, this scoring tool is limited to those admitted with respiratory disease and would need to be combined with other predictive tools to be used to evaluate risk in any child with a suspected infectious illness.

The objective of this research is to develop a prediction model of in-hospital death among children admitted with proven or suspected infectious diseases of any etiology.

## Materials and Methods

### Population

This two-site study was conducted at the Mbarara Regional Referral Hospital (MRRH) and the Holy Innocents Children’s Hospital (HICH), both in Mbarara, Uganda. MRRH is a public hospital funded by the Uganda Ministry of Health and is associated with the Mbarara University of Science and Technology Faculty of Medicine. The pediatrics “Toto” ward admits approximately 5000 patients per year. HICH is a Catholic children’s hospital offering subsidized fee-for-service outpatient and in-patient care in Mbarara and admits approximately 2500 patients annually.

This was a prospective observational study conducted between March 2012 and December 2013 was approved by the institutional review boards at the University of British Columbia (Vancouver, Canada) and the Mbarara University of Science and Technology (Mbarara, Uganda).

### Eligibility

Children aged 6–60 months who were admitted for treatment of a proven or suspected infectious illness were eligible for enrollment. Subjects previously enrolled were excluded (i.e. patients could not be re-enrolled during a subsequent admission). Enrollment was continuous and all children meeting inclusion criteria who were admitted during study working hours or within 8 hours of a study shift were considered eligible. Parents or the legal guardians of eligible children were required to provide written informed consent prior to enrollment.

### Study Procedures

Study enrollment occurred at the time of patient admission. Following enrollment, a research nurse obtained and recorded clinical signs including a 1 minute respiratory rate, blood pressure (automated), axillary temperature, Blantyre coma scale, and using a Phone Oximeter[11], 1 min photoplethysmogram (PPG), blood oxygen saturation (SpO2) and heart rate. Anthropometric data including height, weight, mid-upper arm circumference (MUAC) was also measured and recorded. Anthropometric data collected at enrollment were converted to weight for age, weight for height and height for age z-scores according to the World Health Organization Child Growth Standards.[12] The age corrected heart rate and respiratory rate z-scores were obtained by standardizing the raw measurements using the median and standard deviation values provided by Fleming et al.[13] The age corrected z-scores for systolic blood pressure were calculated using subjects’ height, according to the procedures previously described.[14] To incorporate the clinical belief that both excessively high or low temperature reflect deteriorated health conditions, a transformed temperature was used, which was calculated as 17×log_10_(37.5- temperature) when temperature was less than 37 and as 1.95* temperature -71.3 otherwise. A physiological transformation based on the shape of the relationship between oxygen saturation and virtual shunt [70×log_10_(104-SpO2)-57] was used.[15] This virtual shunt was used as an index of disease severity.

A blood sample was taken for measurement of hemoglobin, HIV and a malaria blood smear (microscopy). HIV status was determined using rapid diagnostic test serial algorithm. All positive tests were confirmed by a separate test. Children under 12 months of age with a positive test were confirmed using PCR. Hemoglobin was measured on a Beckman Coulter Ac.T 5diff Cap Pierce Hematology analyzer.

An interview was conducted with the subject’s parent/guardian and information about previous admissions, distance from health facility, transportation costs, bed-net use, maternal education, maternal age, maternal HIV status, history of sibling deaths and drinking water safety were elicited. Subjects received routine care during their hospital stay and were discharged at the discretion of the treating medical team. The discharge status of all enrolled subjects was recorded as death, referral, discharged alive, and discharged against medical advice. The diagnoses made by the medical team were also recorded.

### Outcomes

The primary outcome was mortality during the course of hospitalization.

### Sample Size

For the derivation of prediction models, standard calculations of sample size do not apply since these calculations do not account for the model selection process (i.e., the optimization to achieve specified sensitivity and specificity cut-offs). One hundred events, corresponding to a total sample of approximately 1000 live-discharges (assuming a mortality rate of 10%, estimated using historical ward data), would be needed to obtain 80% power for ensuring that the lower 95% confidence limit on sensitivity will be at least 75%. An interim analysis of the study showed a mortality rate of approximately 5%. Funding was sufficient to increase enrollment to 1307 subjects.

### Statistical Analysis

All variables were assessed using univariate logistic regression to determine their level of association with the primary outcome. Continuous variables were preferentially analyzed as continuous rather than categorical and were assessed for model fit using the Hosmer-Lemeshow test.[16] Missing data were imputed using the multiple imputation by chained equations method.[17] Following univariate analysis all variables were included in a multivariable logistic model and the primary model was developed using a stepwise selection process. Variables were removed or added individually based on using Aikaike’s Information Criterion (AIC). AIC was used as a summary measure to compare the overall predictive value across the models. This method is considered asymptotically equivalent to cross-validation and bootstrapping.[18,19] All models which having an AIC value within 10% of the lowest value were considered as reasonable candidates. The final selection of a model was judged on model parsimony (the simpler the better), availability of the predictors (with respect to minimal resources and cost), and the attained sensitivity (with at least 50% specificity). These criteria were determined a priori. The area under the curve (AUC) was reported for each model and represents the overall discriminatory ability of the model. All analyses were conducted using R (Vienna, Austria; http://www.R-project.org).

## Results

**Fig 1:**
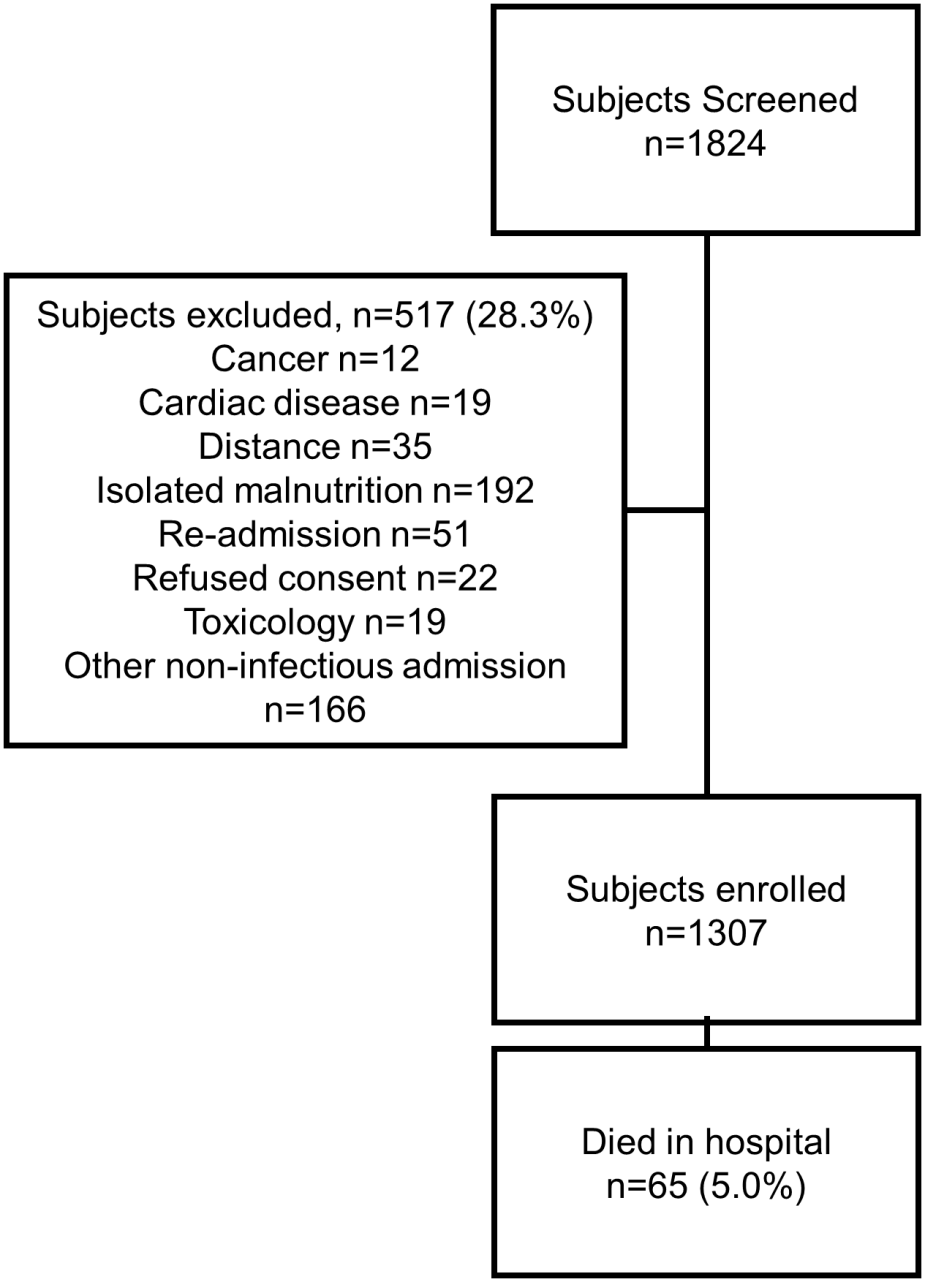
Consort diagram of study flow.

During the study period, 1824 children met the age criteria (six months to five years) and were screened for eligibility. Of these, 517 (28%) were excluded as they were not admitted with a suspected or proven infectious illness. The most common reasons for exclusion included: malnutrition without concurrent infection (n = 192, 37%), already enrolled (n = 51, 10%) living outside of catchment area (n = 35, 7%) and refusal of consent (n = 22, 4%), (Fig 1). In total, 1307 children were enrolled. The median age at admission was 18.2 months (IQR 11.9–33.1) and 717 (54.8%) of subjects were male (Table 1). The proportion of children severely underweight (weight for age z-score less than -3) was 15.7% and 66 (5.1%) of children were HIV positive. The most common clinician assigned diagnoses included clinical malaria (49.7%) pneumonia (31.4%) and gastroenteritis/diarrhea (7.8%).

**Table 1:** Characteristics of Subjects (n = 1307).

General characteristics	Frequency, n (%)
Age 6m–12m	393 (30.1)
Age 12m–24m	402 (30.8)
Age 24m–36m	210 (16.1)
Age 36m–48m	159 (12.2)
Age 48m–60m	142 (10.9)
Female sex	590 (45.1)
Length of stay (days), median (IQR)	3 (2–5)
Discharge AMA	120 (9.7)
Duration of illness < 7 days	841 (64.3)
**Final diagnoses**	
Pneumonia	410 (31.4)
Clinical malaria	659 (50.4)
Parasitemia	434 (33.5)
Gastroenteritis	102 (7.8)
Meningitis	39 (3.0)
**Comorbidities**	
HIV	66 (5.0)
Tuberculosis	23 (1.8)
**Anthropometrics**	
Underweight (WAZ <-2)	372 (28.6)
Severe underweight (WAZ <-3)	206 (15.9)
Wasting (WHZ <-2)	454 (35.3)
Severe Wasting (WHZ <-3)	237 (18.4)
Stunting (HAZ < -2)	368 (28.5)
Severe Stunting (HAZ < -3)	185 (14.3)
MUAC < 125mm	187 (14.5)
MUAC < 115mm	94 (7.3)
**Distance from hospital**	
< 30 minutes	339 (25.9)
30 minutes– 1 hour	290 (22.2)
> 1 hour	678 (51.9)

MUAC: Mid-Upper Arm Circumference

### Mortality

During the course of admission, 65 (5.0%) subjects died in the hospital and 1242 (95%) were discharged alive. The median time to death was 2 days from admission (IQR 1–5). Among those discharged, 120 (9.7%) were discharged against medical advice. Twenty four variables were tested for their univariate association with mortality (Table 2). Blantyre Coma Scale, dichotomized as normal (score of 5) and abnormal (score of < 5), was highly associated with mortality and provided the highest area under the receiver operating characteristic curve, 0.73 (95% CI 0.67–0.79) and an abnormal score being associated with an odds ratio of 11.1 (95% CI 6.59–18.7). All anthropometric variables were associated with mortality during hospitalization. Low weight for age z-scores (underweight) and weight for height/length z-scores (wasting) provided the best discriminatory power for in hospital death with AUC’s of 0.64 (95% CI 0.56–0.71) and 0.63 (95% CI 0.55–0.70), respectively. Both systolic and diastolic blood pressure were associated with mortality, with raw diastolic pressure providing the highest AUC, 0.65 (95% CI 0.58–0.73). Other clinical variables including oxygen saturation, HIV diagnosis, and temperature were also associated with mortality but had lower areas under the ROC curve. Several variables including heart rate, respiratory rate, systolic blood pressure, hemoglobin concentration, and parasitemia were not associated with mortality.

**Table 2:** Univariate analyses of candidate predictor variables for inpatient-mortality.

Variable	n (%)	OR (95% CI)	p-value	AUC ROC (95% CI)
Age (months)	1306 (99.9)	1.00 (0.99–1.02)	0.85	0.51 (0.44–0.58)
Sex (female)	1307 (100)	0.96 (0.58–1.58)	0.87	0.51 (0.44–0.57)
MUAC	1293 (98.9)	0.98 (0.96–0.99)	<0.001	0.60 (0.53–0.68)
Weight	1300 (99.5)	0.89 (0.81–0.97)	0.008	0.59 (0.52–0.67)
Weight-age z-score	1299 (99.4)	0.75 (0.65–0.87)	<0.001	0.64 (0.56–0.71)
Weight-length z-score	1282 (98.1)	0.80 (0.72–0.89)	<0.001	0.63 (0.55–0.70)
Height-age z-score	1286 (98.4)	0.90 (0.80–1.00)	0.05	0.57 (0.50–0.64)
BMI-age z-score	1282 (98.1)	0.81 (0.71–0.92)	<0.001	0.62 (0.54–0.69)
Heart rate	1306 (99.9)	0.99 (0.98–1.00)	0.04	0.55 (0.47–0.63)
Heart rate z-score	1305 (99.8)	0.87 (0.76–1.00)	0.05	0.53 (0.45–0.62)
Resp. rate	1306 (99.9)	1.01 (1.00–1.03)	0.17	0.56 (0.49–0.64)
Resp. rate age z-score	1305 (99.8)	1.06 (1.00–1.12)	0.06	0.55 (0.48–0.63)
SBP	1298 (99.3)	0.98 (0.96–0.99)	0.01	0.60 (0.53–0.68)
SBP z-score	1277 (97.7)	0.84 (0.70–1.00)	0.04	0.59 (0.51–0.67)
DBP	1298 (99.3)	0.96 (0.94–0.98)	<0.001	0.65 (0.58–0.73)
Transformed SpO2	1291 (98.8)	1.03 (1.01–1.05)	<0.001	0.59 (0.50–0.68)
Temperature–raw	1307 (100)	0.68 (0.57–0.80)	<0.001	0.61 (0.54–0.68)
Temperature–transformed	1307 (100)	1.05 (0.93–1.18)	0.47	0.50 (0.43–0.57)
Blantyre Coma Scale^1^	1307 (100)	0.09 (0.05–0.15)	<0.001	0.73 (0.67–0.79)
Hemoglobin (g/dL)	1299 (99.4)	0.94 (0.87–1.02)	0.16	0.56 (0.48–0.64)
Parasitemia (ref: neg)	1297 (99.2)	0.65 (0.36–1.16)	0.14	0.54 (0.49–0.60)
SR Maternal HIV (ref: neg)	1087 (83.2)	1.91 (0.93–3.95)	0.08	0.55 (0.48–0.61)
HIV status (ref: neg)	1263 (96.6)	5.02 (2.22–11.38)	<0.001	0.58 (0.51–0.64)

^1.^ dichotomized in to normal vs abnormal as too few with score less than 4

SBP: Systolic Blood Pressure, DBP: Diastolic Blood Pressure, SR: Self Report

### Multivariate Prediction Model

Three models were developed for prediction of mortality. A primary model was developed using any of the available variables. Subsequent models were derived which selectively excluded certain variables from the primary model to ensure that prediction could be possible in the absence of certain variables which may not be available at all centers.

**Fig 2:**
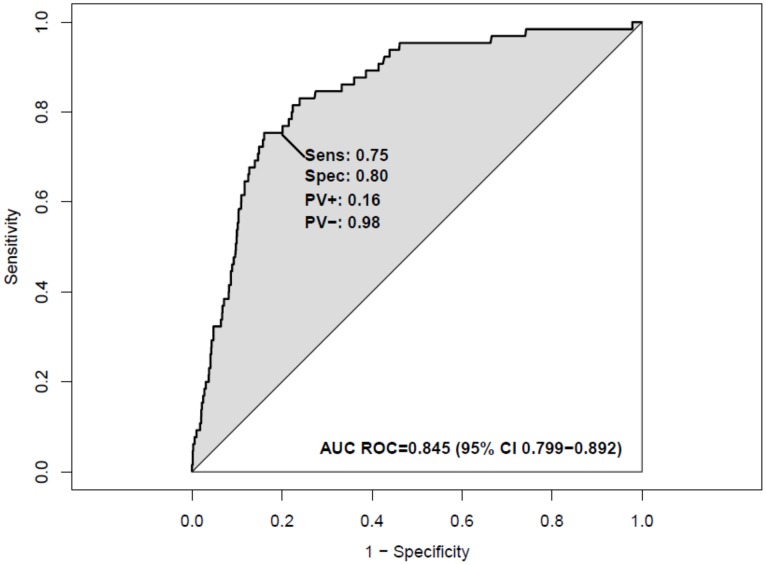
Primary model receiver operating curve characteristics.

The first model included weight for age z-score, Blantyre coma scale and HIV status. The model equation was: logit [Pr (In-patient mortality)] −1.78+(−0.26; weight for age z-score) −2.50 (normal Blantyre coma scale) +1.32 (positive HIV diagnosis) and the area under the receiver operator characteristic curve was 0.85 (95% CI 0.80–0.89). At a probability cut-off of 0.030, this model had a sensitivity of 0.83 (95% CI 0.74–0.92) and a specificity of 0.76 (95% CI 0.73–0.78). We would expect the positive predictive value to be 0.15 (95% CI 0.11–0.19) and the negative predictive value to be 0.99 (95% CI 0.98–1.00) (Tables 3 and 4, Fig 2).

**Table 3:** Model Characteristics.

Model	AUC (95% CI)	Sens. (95% CI)	Spec. (95% CI)	PPV (95% CI)	NPV (95% CI)
1	0.85 (0.80–0.89)	0.83 (0.74–0.92)	0.76 (0.73–0.78)	0.15 (0.11–0.19)	0.99 (0.98–1.00)
2	0.84 (0.79–0.89)	0.80 (0.70–0.90)	0.76 (0.74–0.79)	0.15 (0.11–0.19)	0.99 (0.98–1.00)
3	0.82 (0.72–0.91)	0.82 (0.72–0.91)	0.71 (0.68–0.73)	0.13 (0.10–0.16)	0.99 (0.98–0.99)

PPV: Positive Predictive Value, NPV: Negative Predictive Value

**Table 4:** Models Developed for Prediction of In-patient Mortality.

Variable	Regression Estimate	p-value	OR (95% CI)
**Model 1 –Primary model, Intercept = -4.280**			
Abnormal BCS	2.51	<0.001	12.30 (7.10–21.30)
Positive HIV diagnosis	1.32	0.007	3.74 (1.46–9.57)
Weight-age z-score	-0.25	0.002	0.78 (0.66–0.91)
**Model 2 –Model derived without weight for age z-score, Intercept = -0.523**			
Abnormal BCS	2.54	<0.001	12.68 (7.31–22.01)
Positive HIV diagnosis	2.27	0.006	3.79 (1.48–9.71)
MUAC (mm)	-0.03	0.002	0.98 (0.96–0.99)
**Model 3 –Model derived without HIV and weight for age z-score, Intercept = 0.303**			
Abnormal BCS	2.47	<0.001	11.78 (6.90–20.13)
MUAC (mm)	-0.03	<0.001	0.97 (0.96–0.99)

BCS: Blantyre Coma Scale, MUAC: Mid-Upper Arm Circumference

Model 2 replaced weight for age z-score in model 1 with mid-upper arm circumference, a variable that is more easily obtainable, especially in poorly resourced areas. The area under the ROC curve was 0.84 (95% CI 0.79–0.89). At a probability cut-off of 0.030 this model had a sensitivity of 0.80 (95% CI 0.70–0.90) and specificity of 0.76 (95% CI 0.74–0.79) and we would expect the positive predictive value and negative predictive value of 0.15 (95% CI 0.11–0.19) and 0.99 (95% CI 0.98–1.00), respectively, in a population similar to the derivation cohort (Tables 3 and 4).

Model 3 included MUAC and Blantyre coma scale and excluded HIV. At a probability cut-off of 0.30 this model had a sensitivity of 0.82 (95% CI 0.72–0.91) and a specificity of 0.71 (95% CI 0.68–0.73). The expected positive and negative predictive values would be 0.13 (95% CI 0.10–0.16) and 0.99 (0.98–0.99) (Tables 3 and 4).

## Discussion

This study represents a systematic approach to creating an in-hospital mortality prediction tool for the under-five pediatric population admitted with an infectious illness in a resource constrained environment. The model developed is parsimonious, using only age, weight, Blantyre coma scale and HIV status to determine the probability of in-hospital mortality. Variables used in the development of the prediction model included only those thought to be both easily and reliably obtainable in most resource constrained contexts. Alternate models were developed incorporating different elements to ensure prediction would be possible in situations where certain variables may not be available.

In the context of limited resources, rapid risk determination is of critical importance. The implementation of an improved triage and training system (ETAT) alongside improvements in patient flow was shown to decrease in-patient mortality, especially early in-patient mortality.[20] Prediction models such as the one derived in this study could be used alongside a comprehensive strategy such as ETAT to improve care at the point of admission and focus human (eg. nursing) and clinical (eg. oxygen) resources on those children at highest risk of mortality. The determination of risk could play a unique role in sub-Saharan Africa where critically ill children admitted to lower level health centers require sufficient time to travel to referral hospitals. Further, as referral decisions are often made by non-physician health care providers, decision tools such as these could offer substantial aid with minimal training to ensure that those at high risk of mortality are referred. Using a risk-cut-off of 0.30, the referral population would have a mortality risk of 15% (95% CI 11% - 19%) compared to a mortality risk of 1% (95% CI 0% - 2%) in those not referred, if similar to the admitted sample in this study. Future research must, however, examine the effect of transporting critically ill children as identified with these models as transportation itself may confer its own risks.

Other models developed for mortality prediction in settings without resource constraints have been evaluated in resource constrained settings have been shown to not perform adequately. A critical difference in the populations in whom models such as PRISM were derived is the much lower prevalence of moderate and severe malnutrition. In this present study an anthropometric measure played a crucial role in all derived models, providing a majority of the discriminatory power in final model. Malnutrition as an independent and critical contributor to infectious disease morbidity and mortality cannot be over emphasized and should be considered as an important component of the evaluation of any infectious illness. In addition to malnutrition, an abnormal Blantyre coma scale was also of critical importance, proving more discriminatory than vital signs in predicting mortality.

Although the Integrated Management of Childhood Illness algorithm was not designed as a prediction tool, it does provide referral criteria, listed as danger signs. A recent study from Tanzania evaluated the predictive utility of these criteria for in-hospital mortality. Among 387 children aged two months to five years, and an overall mortality rate of 7.4%, one or more IMCI danger sign had a sensitivity of 72% (95% CI 56% - 88%) for predicting in-hospital death and would identify 38% of subjects as high-risk.[21] Using a cut-off of 0.30, our model, using fewer and more reliably obtained variables, has a higher sensitivity of 0.83 (95% CI 0.74–0.92) and would only identify approximately 25% as high-risk, allowing for a more efficient utilization of resources.

This study was limited by fewer cases of the primary outcome (in-hospital mortality) than was initially anticipated. Although designed to derive prediction models using 100 outcomes, this enrolled only 65 children who died in-hospital. Despite the relatively low number of outcomes our primary model had an AUC of the receiver operating characteristic curve of 0.85 with the lower limit being 0.80, highlighting the excellent discriminating ability of the final model and the utility of each of the predictive variables in the model. A further limitation is that currently it is not known whether the identification of high-risk children can actually result in saved lives, although it would be difficulty to justify not acting on such information. Another limitation of this study are the mathematical calculations involved with the use of the proposed models. To address this important limitation our research group has developed and evaluated a mobile application of this model and one for post-discharge mortality. Finally, the lack of external validity limits widespread use and scaling of this model.[22] Wider validation studies must be conducted prior to the implementation of this model in any setting.

Although a major strength of this study was its development using more than one hospital, these results require confirmation at other hospitals in other resource constrained countries for further calibration prior to recommending their uptake. This is of particular importance if used to aid in referral decision making at lower level health centers, as admission criteria are certainly different than in larger hospitals producing a unique population of children. These models could also aid in the development of a standardized in-patient mortality prediction score could also be helpful in the designing of clinical trials of interventions in resource constrained settings.

In conclusion, a parsimonious prediction tool using easily collected predictors can be used to efficiently predict in-hospital mortality. Further research to externally validate this model is required prior to widespread implementation.
